# In-flight cosmic radiation doses along the current alternative routes between Tokyo and Frankfurt

**DOI:** 10.3389/fpubh.2026.1968494

**Published:** 2026-10-06

**Authors:** Hiroshi Yasuda, Kazuaki Yajima, Tatsuhiko Sato

**Affiliations:** 1 Hiroshima University, Hiroshima, Japan; 2 National Institutes for Quantum Science and Technology, Chiba, Japan; 3 Japan Atomic Energy Agency, Tokai, Ibaraki, Japan

**Keywords:** alternative route, aviation, cosmic radiation, in-flight dose, JISCARD, NRF31

## Abstract

**Background:**

Calculation-based dose assessment for aircraft crew is required worldwide by authorities, as cosmic radiation dose rates at aviation altitudes are substantially higher than at sea level. The accuracy of the calculated in-flight dose onboard aircraft (“route dose” hereafter) should be confirmed periodically, particularly when the flight route is significantly changed.

**Methods:**

We focused on the long-haul flights between Tokyo and Frankfurt, which have recently undergone significant changes owing to the geopolitical conflicts between Ukraine and the Russian Federation and in the Middle East. The route doses for a round trip in February 2026 under solar maximum conditions were calculated using actual flight path records and the JISCARD EX route-dose calculation program. Calculated route doses were compared with measurements obtained using an electronic neutron dosimeter to check the consistency.

**Results:**

The in-flight effective doses were estimated to be 51 μSv for the north route from Tokyo to Frankfurt and 30 μSv for the south route from Frankfurt to Tokyo. In-flight measurements using the electronic neutron dosimeter showed trends consistent with calculated ambient dose equivalents of heavy charged particles. The effective doses for the north and south routes with a specified constant cruising altitude of 40,000 ft. increased by 10% and decreased by 24%, respectively, compared to the great-circle route doses.

**Conclusion:**

The findings in this study indicate that recent route changes have substantially altered the pattern of cosmic radiation exposure onboard aircraft. While changes in the total in-flight dose may be generally small and annual doses of aircraft crew would decrease if their onboard working hours are carefully managed, the potential risk of elevated radiation exposure due to a solar particle event is higher on the north route.

## 1 Introduction

The level of cosmic radiation exposure at aviation altitudes (25,000–40,000 ft. or 8–13 km) is notably higher than at sea level. Accordingly, aircraft crew and some passengers who frequently fly can receive relatively high doses that can reach several mSv per year (1). Based on this fact, the International Commission on Radiological Protection (ICRP) stated in the 1990 recommendations that in-flight exposure of aircraft crew should be managed as occupational radiation exposure (2). This implies that employers of aircraft crew are responsible for implementing the necessary measures to protect their employees. The 1990 recommendations were confirmed in the subsequent basic recommendations in 2007 (3), which states that cosmic radiation exposure is considered an existing exposure situation because the source exists prior to any protection decisions. In addition, the ICRP recently recommended that some frequent flyers who often travel using commercial aircraft should be managed in a manner similar to those occupationally exposed on a case-by-case basis, even though their exposure is classified as the public exposure (1).

Following the recommendations of the ICRP, the European Union (EU) introduced a Basic Safety Standards Directive (BSS), which included exposure to natural sources of ionizing radiation, including cosmic radiation, as occupational exposure for aircraft crew (4, 5). The EU Council Directive has been incorporated into the laws and regulations of many EU Member States and subsequently into the aviation safety standards and procedures of the European Aviation Safety Agency. Other countries, such as Canada and Japan, have also issued advisories to their airline industries to manage aircraft crew exposure (6, 7). The International Atomic Energy Agency (IAEA) has provided basic policies and practical guidelines in the IAEA Safety Standards Series (8, 9), and the International Organization for Standardization (ISO) has provided technical guidance for cosmic radiation dosimetry in civilian aircraft (10–13). These standards recommend that the accuracy of the computer code used for calculating in-flight cosmic radiation doses should be periodically validated with in-flight measurements based on the ambient dose equivalent at a depth of 10 mm, H*(10), using a detector that is carefully calibrated in suitable reference fields, since the detector response to the complex radiation field at aviation altitudes can vary significantly depending on its structure and detection mechanism.

However, it is difficult to perform comprehensive measurements using large-scale devices on commercial flights because of cost and safety constraints. Therefore, past in-flight measurements using relatively large detectors have been performed with support of airline companies (14–18). Considering this difficulty, it would be beneficial to use portable electronic dosimeters that can automatically record dose rate changes during a long flight, although many of the commercial dosimeters designed for monitoring photons and electrons do not show sufficient responses to the cosmic radiation field at aviation altitudes (19, 20), where energetic neutrons and protons contribute significantly to the effective dose.

Verifying calculated in-flight doses based on the actual flight paths is particularly important when a long-haul flight route deviates significantly from a great-circle route, which represents the shortest route on a sphere. Thus, we focused on long-haul flights between Asia and Europe because their routes have recently undergone large changes owing to the conflicts between Ukraine and Russia and in the Middle East, and examined the effects of these air-route changes on cosmic radiation exposure in civilian aircraft for a round trip from Tokyo to Frankfurt through the analysis of measured and calculated data. Although a preceding simulation study examined the effect of the route change for Paris–Tokyo flights based on the calculations of ambient dose equivalents using their own simulation platform (ATMOS CORE) (21), the temporal profiles of estimated doses have not been compared with onboard measurements. In addition, the previous study did not present effective doses, although the demonstration of this quantity is important for further discussions on the radiological protection of aircraft crew and frequent flyers (1). Therefore, we attempted to confirm the consistency of calculated H*(10) profiles using the measurement data obtained with a commercial electronic neutron dosimeter onboard aircraft and discussed the effects of the recent route changes on the in-flight effective doses.

## 2 Methods

### 2.1 Calculation of route doses

In-flight cosmic radiation doses were calculated for round-trip flights from Tokyo, Japan to Frankfurt, Germany in February 2026, which was in the solar maximum phase, using the actual flight path records provided by an airline in Japan. It should be noted that the specific flight dates or flight numbers are withheld under a confidentiality agreement with the airline, and the disclosure of these data requires a prior approval from the airline. The outbound flight (from Tokyo to Frankfurt) took a north polar route, and the inbound flight (from Frankfurt to Tokyo) took a south alternative route, as shown in Figure 1A. For the selected flights with these alternative routes in February 2026, the temporal changes in the recorded flying altitude are shown in Figure 1B. The ascent and descent times for the north route were 23 and 20 min, respectively, and those for the south route were 26 and 20 min, respectively. Figure 1C shows the temporal changes in the geomagnetic cut-off rigidity for both flights; cut-off rigidity indicates the Earth’s geomagnetic shielding against primary cosmic radiation; more specifically, this parameter means the minimum momentum-to-charge ratio required for a charged cosmic-ray particle to penetrate the Earth’s magnetic field, ranging from approximately 0 GV at the poles to nearly 15 GV at the equator. To discuss the effects of the air-route changes, route doses were calculated for the great-circle route that was taken before the eruption of the recent conflicts between Ukraine and the Russian Federation and in the Middle East. As seen in Figure 1A, the great-circle route (dotted line) is notably shorter than the alternative routes.

**Figure 1:**
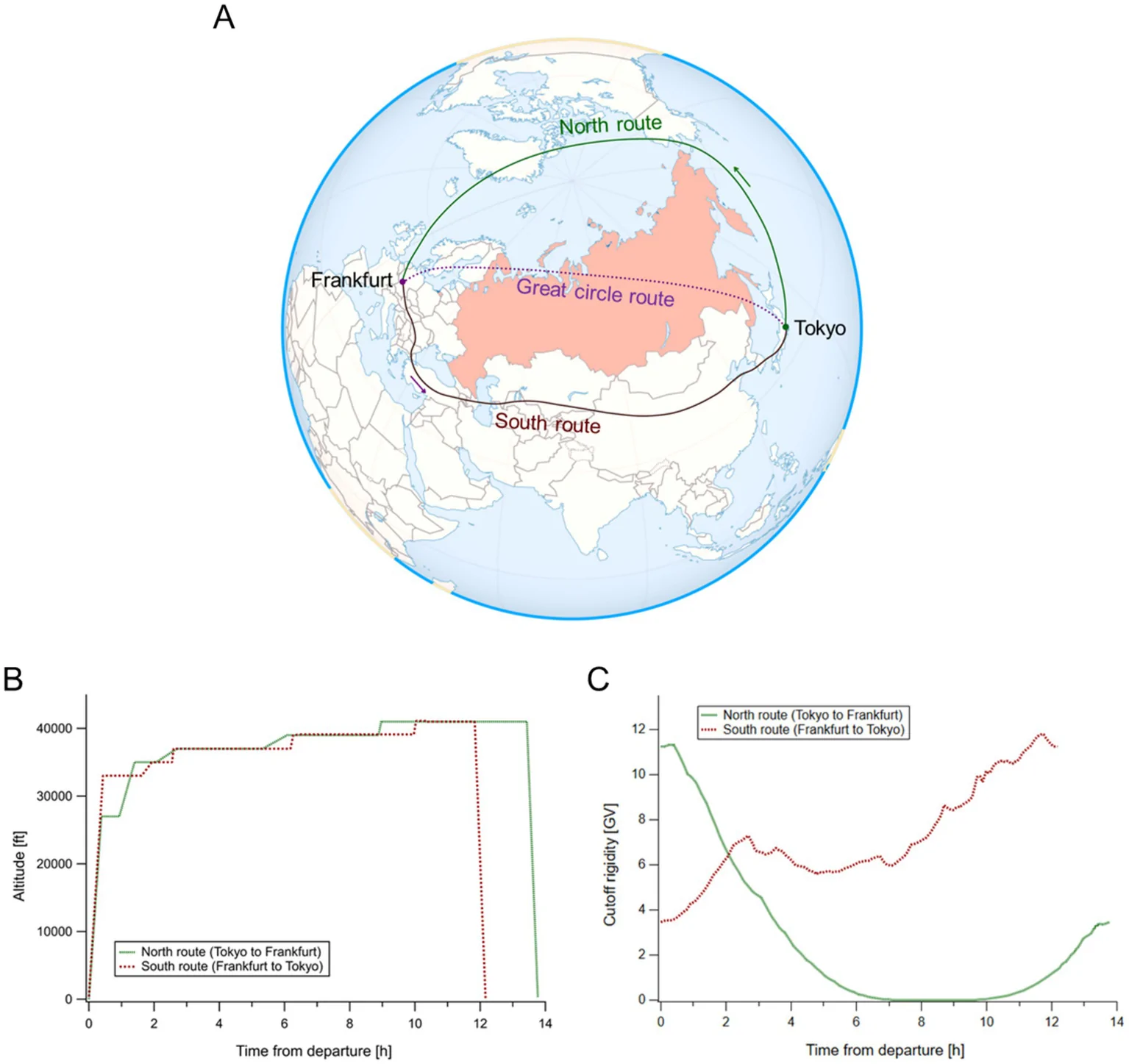
The flight routes investigated in this study. **(A)** Illustration of the outbound and inbound flight routes for the round trip from Tokyo to Frankfurt in February 2026, compared to the previous great-circle route. **(B,C)** Temporal profiles in the recorded flying altitude and geomagnetic cut-off rigidity, respectively, for both routes.

The in-flight effective dose and 1 cm ambient dose equivalent, H*(10), along both flight routes were calculated using the route-dose calculation program “JISCARD EX,” which has been used for the management of cosmic radiation exposure of aircraft crew of major airline companies in Japan over the past 20 years (7, 22). JISCARD-EX was originally developed by the authors as a program package of “JISCARD” (23) in 2007 and has been improved through the incorporation of an analytical model “PARMA,” which can quickly calculate the cosmic radiation dose rates in the atmosphere (24, 25). In this study, we used the 2016 version of PARMA, which became available to external users in early 2016 following the publication of the 2015 article (25) describing the updated model specifications. The PARMA calculates terrestrial cosmic ray fluxes at any altitude up to 20 km based on the simulation results of extensive air shower (EAS) induced by galactic cosmic rays. The model comprises several theoretical and empirical equations with free parameters, of which the numerical values were determined from the least–squares fitting of the EAS simulation results obtained using the Particle and Heavy Ion Transport Code System (PHITS) (26). The model can provide the fluxes of major cosmic-ray components: protons, helium ions, muons, electrons, positrons, and photons in the energy range from 1 MeV to 100 GeV (per nucleon for He ions) and neutrons in the range from 0.01 eV to 100 GeV.

The accuracies of the aviation doses calculated by JISCARD EX were partially validated through comparisons with in-flight measurements (17, 18, 27, 28). In addition, dose calculations made by JISCARD EX were compared with those by other international codes, such as EPCARD, AVIDOS, SIEVERT, and PCAIRE, and the overall agreement was better than ±20% (29, 30), which is considered satisfactory for the aim of radiological protection based on effective dose evaluation. In this study, we used the JISCARD EX incorporating the 2016 version of PARMA, which is explained in detail in the article published in 2015 (25). Effective doses were calculated using a monthly averaged solar activity level (force field potential, FFP) and the up-to-date radiation and tissue weighting factors provided by ICRP in their 2007 recommendations (3).

### 2.2 In-flight measurements using an electronic neutron dosimeter

To check the consistency of calculated route doses, in-flight radiation exposure during the round-trip flights was continuously monitored using an electronic personal neutron dosimeter (NRF31, Fuji Electric Co., Ltd., Tokyo) (Figure 2A). The NRF31 is a battery-powered portable dosimeter with the dimensions of 60 × 78 × 33 mm^3^ and a weight of approximately 120 g. This dosimeter can continuously work without a battery change for nearly 2000 h under normal indoor conditions (31, 32). One dosimeter was placed in a hand luggage and placed under a cabin seat in the middle row of an aircraft during round-trip flights. Hourly rates of the personal dose equivalent at a depth of 10 mm, *Hp*(10), of neutrons were saved at 10 min intervals, and the time-sequential cumulative doses were read out via infrared (IR) communication using an exclusive software on a Windows PC.

**Figure 2:**
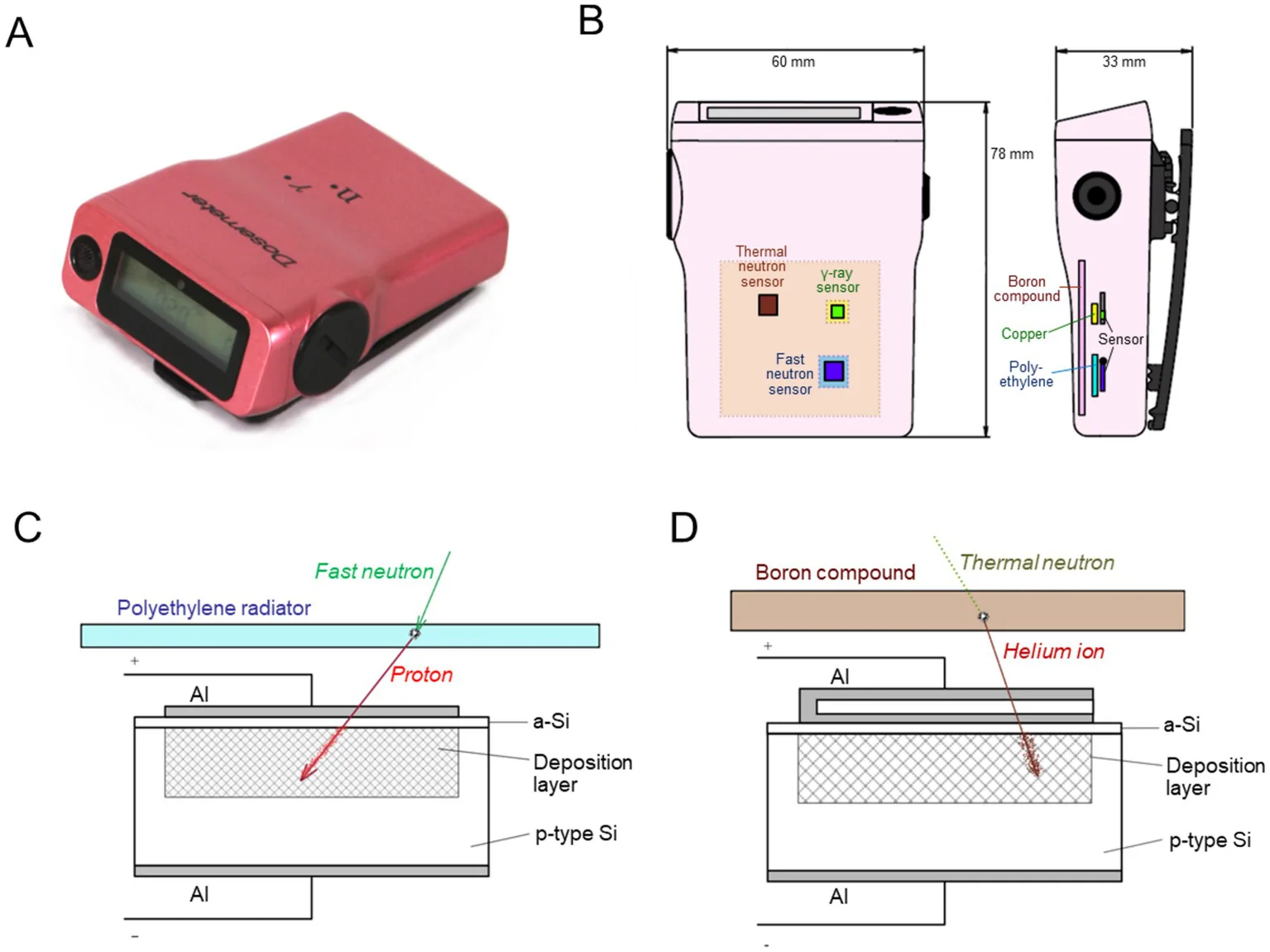
The electronic neutron dosimeter (NRF31) used for in-flight measurements. **(A)** Photograph of the dosimeter. **(B)** Sensor dimensions and detector arrangement. **(C,D)** Schematic structures of the fast neutron sensor and thermal neutron sensor, respectively.

The positions of the neutron sensors in the NRF31 dosimeter are indicated in Figure 2B. The neutron *Hp*(10) data were obtained using two types of silicon semiconductors: fast-neutron (>1 MeV) and slow-neutron (<1 MeV) sensors. The fast neutron sensor was a 10 × 10 mm^2^ p-type silicon crystal deposited with an amorphous silicon hydride (Figure 2C), and the slow neutron sensor was a 10 × 10 mm^2^ p-type silicon crystal deposited on an aluminum electrode covered by a boron compound layer (Figure 2D). This dosimeter does not have the issue of radio wave interference, which can be a concern for use in commercial aircraft. The neutron energy response of the NRF31 was calibrated for monoenergetic neutrons from 250 keV to 15 MeV and confirmed that it agreed with the fluence-to-dose-equivalent conversion coefficient provided by the ICRP (33) within ±50% (32).

## 3 Results

### 3.1 Calculated in-flight effective doses

Calculated in-flight effective doses and H*(10) values for the round-trip flights from Tokyo to Frankfurt in February 2026 are summarized in Table 1. The effective doses were estimated to be 51 μSv for the outbound north-route flight and 30 μSv for the inbound south-route flight. The trends in in-flight H*(10) rates were consistent across both flights, although the H*(10) values were 11–13% higher than the corresponding effective doses.

**Table 1:** In-flight effective doses, *E*, and ambient dose equivalents, H*(10), calculated from actual flight path records for the round-trip flights between Tokyo and Frankfurt in February 2026.

Route	Flying time [hh:mm]	Effective dose (E) [μSv]	H*(10) [μSv]	H*(10)/E ratio
North (Tokyo -> Frankfurt)	13:46	50.9	57.4	1.13
South (Frankfurt -> Tokyo)	12:10	30.2	33.5	1.11
Round trip	25:56	81.1	90.9	1.12

Temporal changes in the calculated effective dose rates during both flights are shown in Figure 3A. The dose along the south route was notably lower than that along the north route. Figures 3B,C show the cumulative effective doses of the major components of cosmic radiation at aviation altitudes for the north and south route flights, respectively. The radiations were classified here into three groups: neutrons, HCPs (protons and helium ions), and other light components (muons, electrons, positrons, and photons). The total effective dose in the north route (from Tokyo to Frankfurt) was calculated to be 51 μSv, and that in the south route (from Frankfurt to Tokyo) was 30 μSv. Although the most contributing component was neutrons in both routes on the effective-dose basis, the fraction of neutrons was higher in the north route than that in the south route, as expected from the difference in the cut-off rigidity profiles (Figure 1C).

**Figure 3:**
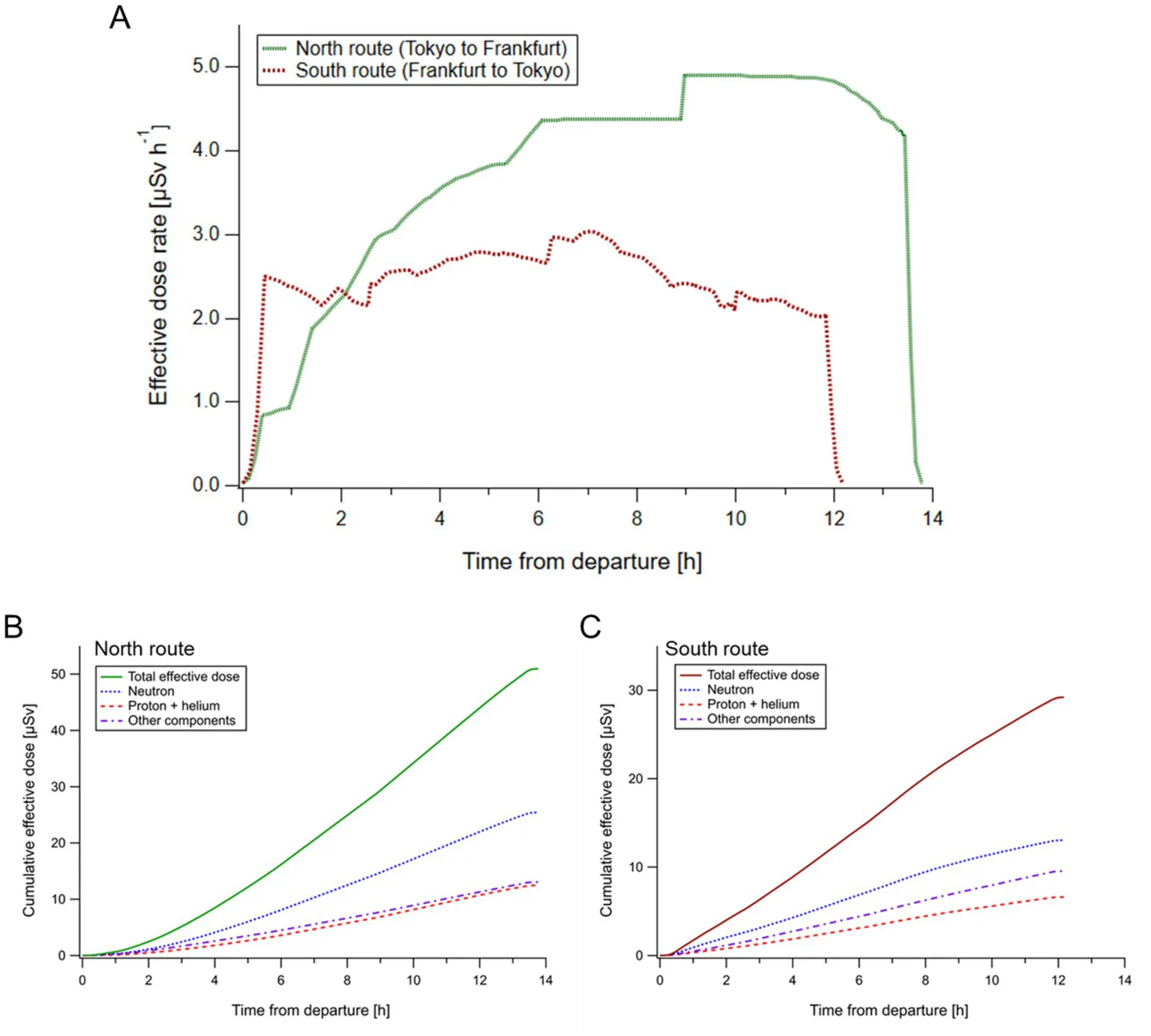
Calculated effective doses for the Tokyo–Frankfurt flights in February 2026. **(A)** Temporal changes in the calculated effective dose rates for the north and south route flights. **(B,C)** Temporal changes in the calculated cumulative effective doses by components (total, neutrons, HCPs, and others) for the north and south route flights, respectively. Note that the plots of total effective dose for the north route **(B)** and south route **(C)** were colored green and wine red, respectively, for consistency with the route colors in Figure 1A.

### 3.2 Consistency checks of the calculated route doses

According to the ISO recommendations (10–13), we attempted to check the consistency of the calculated H*(10) values by comparing them with the dosimeter-indicated *Hp*(10) values over the round-trip flights from Tokyo to Frankfurt in February 2026. According to the detection mechanism of this electronic neutron dosimeter (NRF31) (Figures 2C,D), it was expected that certain fractions of heavy charged particles (HCPs), that is, protons and helium ions, could be counted as neutron-induced HCPs, which would cause a higher value of dosimeter-indicated neutron dose. This expectation was confirmed, as shown in Figures 4A,B. The NRF31 exhibited neutron dose values approximately 3.9 times higher than the calculated H*(10) values for the north route and 3.3 times higher for the south route, whereas the dosimeter-indicated values were approximately 25 times higher than the H*(10) values of HCPs in both cases. These findings implied that the NRF31 overestimated neutron doses owing to its sensitivity to the HCPs of cosmic radiation at aviation altitudes, even though the calculated H*(10) values for neutrons were markedly higher than those for HCPs, as shown in Figures 4C,D. Calculation results also demonstrated that the relative contribution of HCPs to the dosimeter-indicated value was higher for the north route than that for the south route, as implied by the difference in the cut-off rigidity between two routes (Figure 1C); the HCP fractions of the total H*(10) were 16.0 and 13.8% for the north and south route, respectively. The fraction of helium ions to H*(10) of HCPs was approximately 0.13%.

**Figure 4:**
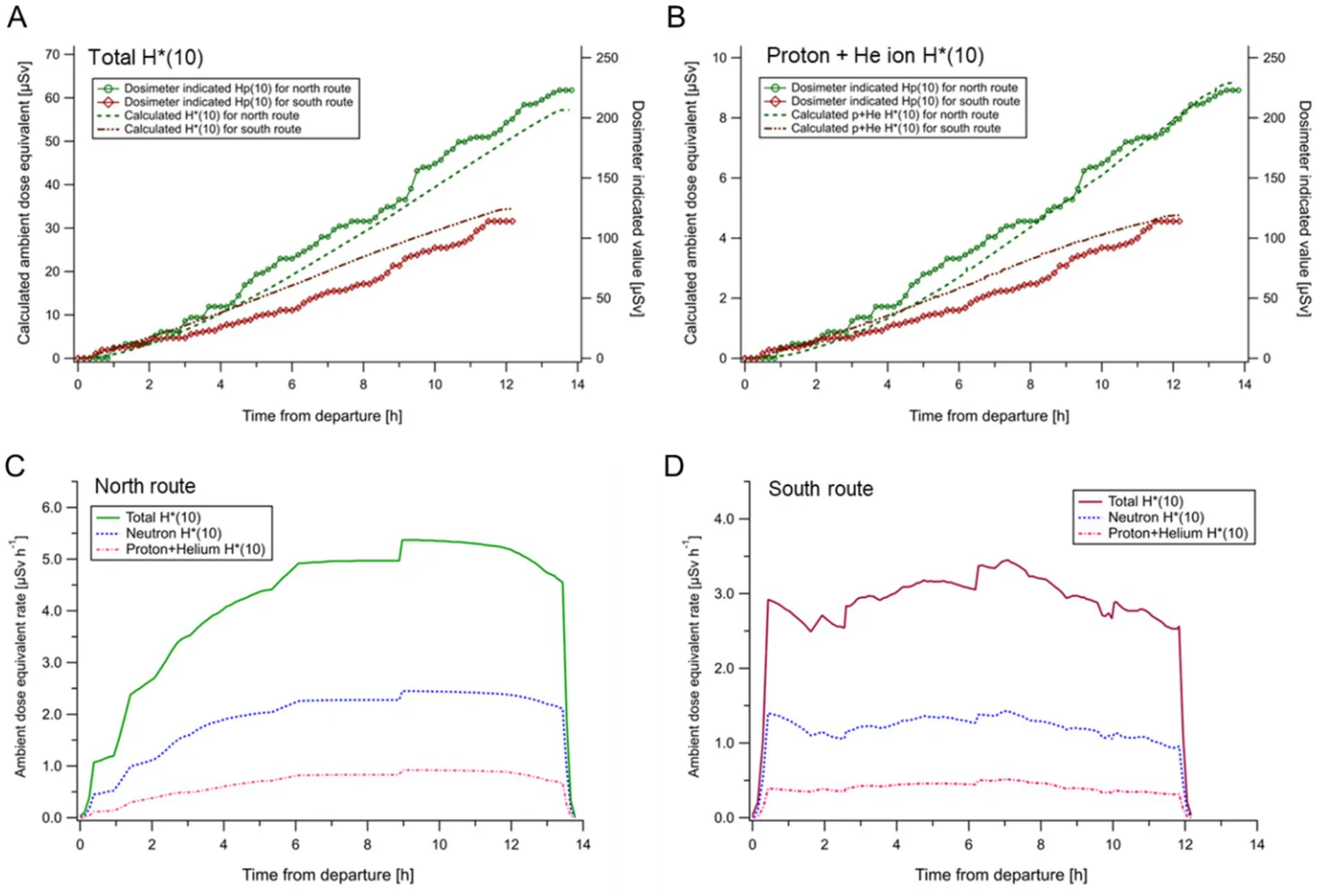
Comparison between calculated H*(10) and dosimeter-indicated values. **(A,B)** Comparisons of the dosimeter-indicated values recorded by the NRF31 dosimeter and the calculated cumulative in-flight H*(10) values for all components and those for HCPs (protons and helium ions), respectively. **(C,D)** Temporal changes in calculated H*(10) rates by component (total, neutrons, and HCPs) for the north and south route flights, respectively. Note that the y-axis ranges for dosimeter-indicated values in panels **(A,B)** are 3.6-fold and 25-fold greater than those for calculated total and HCP H*(10) values, respectively. The plots of total H*(10) for the north route **(C)** and south route **(D)** were colored green and wine red, respectively, for consistency with the route colors in Figure 1A.

### 3.3 Comparison with great-circle route doses

The route doses were also calculated for virtual flights using the previous great-circle route (Figure 1A) in February 2026 and compared them with those using the current alternative routes. In this comparative analysis, a constant cruising altitude of 40,000 ft. (approximately 12 km) was assumed for all flights while maintaining the same ascending and descending times to eliminate the effects of flying altitudes, that is, atmospheric pressures. Assuming a constant cruising altitude of 40,000 ft. was deemed reasonable based on actual flight path records over the past 20 years (which cannot be disclosed due to a confidentiality agreement with the airline) for three main reasons: Firstly, some long-haul flights from Japan to Europe occasionally maintained a constant cruising altitude between 37,000 ft. and 41,000 ft. Second, the past records also demonstrate that altitude profiles of a given flight vary significantly over time due to operational factors, making it difficult to uniquely define a representative altitude profile. Third, the effective dose ratios between different routes (i.e., different cut-off rigidity profiles) were not significantly affected by cruising altitudes within the range between 37,000 ft. and 40,000 ft. (22). The great-circle flight duration data were obtained from the flight schedule published by the airline in 2019, prior to the disruptions from the COVID-19 pandemic as well as the Ukraine-Russia conflict.

Figures 5A,B present the temporal changes in the cutoff rigidity and calculated effective dose rate, respectively, for round-trip flights along the great-circle route from Tokyo to Frankfurt. The changes in both the cut-off rigidity and effective dose were symmetrical between the outbound and inbound flights, although there was a slight difference in flight duration.

**Figure 5:**
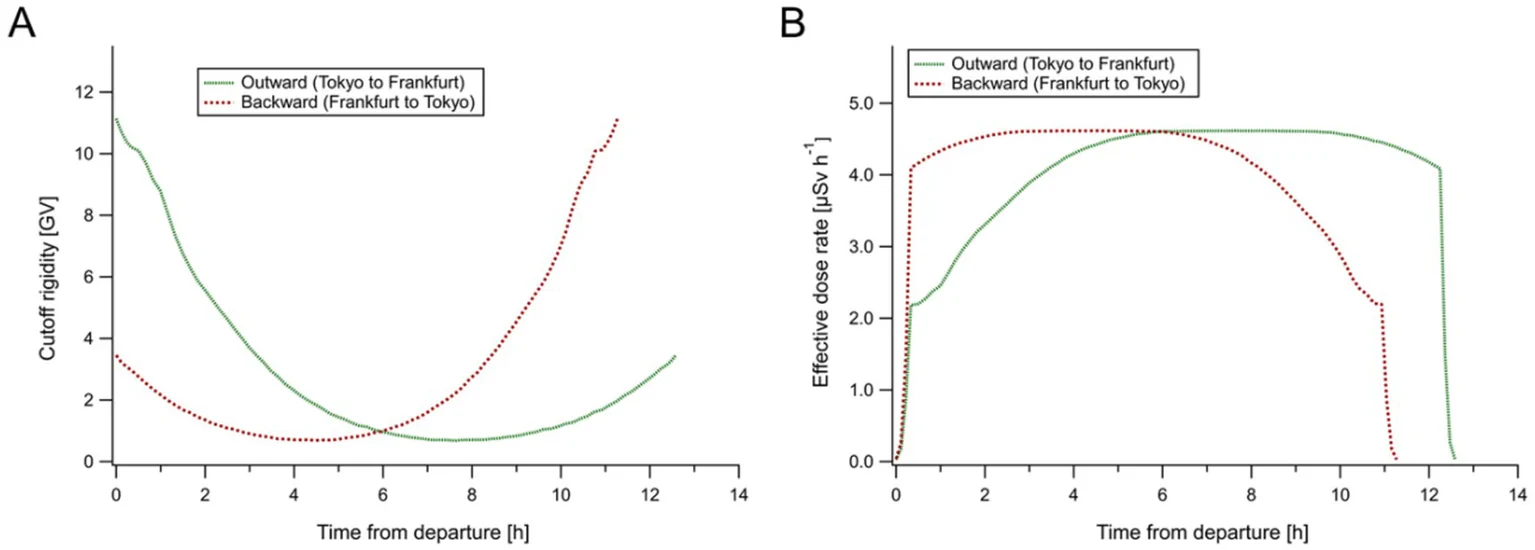
Conditions and results of the effective dose calculation for the Tokyo–Frankfurt round-trip flights along the great-circle route in February 2026. **(A)** Temporal profiles of geomagnetic cut-off rigidity for the great-circle route flights. **(B)** Corresponding temporal profiles in the calculated effective dose rates.

The results of effective dose calculations are summarized in Table 2. The great-circle route doses for outbound and inbound flights with a specified constant cruising altitude (40,000 ft) were calculated as 50 μSv and 45 μSv, respectively. On the other hand, the route doses calculated for the alternative north and south routes (i.e., cut-off rigidity profiles shown in Figure 1C) with the same cruising altitude were calculated as 55 μSv and 34 μSv, which correspond to relative changes of 10% and −24%, respectively, from those for the great-circle routes. These dose levels were slightly higher than those calculated from actual flight path records (Figure 3) because of the assumption of a higher altitude (40,000 ft). Regarding the total effective dose for the round trip, the dose for the alternative routes (89 μSv) was 6% lower than that for the great-circle route (95 μSv). The dose per flight hour for the flight along the alternative north route was virtually unchanged (+0.5%) from that for the great-circle route, whereas the dose per flight hour for the alternative south route decreased notably by 30%. Consequently, the round-trip dose per flight hour for the current alternative routes showed a 14% reduction relative to that for the great-circle route.

**Table 2:** In-flight effective doses calculated for the conventional great-circle routes and current alternative routes with a specified constant cruising altitude of 40,000 ft (approximately 12 km) between Tokyo and Frankfurt in February 2026.

Direction	Flight route	Flying time [hh:mm]	Effective dose [μSv]	Dose per flight hour [μSv h^−1^]
Tokyo -> Frankfurt	Great circle	12:35	50.1	3.98
	North	13:46	55.0	4.00
Frankfurt -> Tokyo	Great circle	11:16	44.7	3.97
	South	12:10	34.0	2.80
Round trip (Tokyo – Frankfurt)	Great circle	23:51	94.8	3.98
	North + South	25:56	89.0	3.43

## 4 Discussion

In this study, we investigated the effects of recent changes in flight routes between Asia and Europe, which were caused by the conflicts between Ukraine and Russia and in the Middle East, on cosmic radiation exposure onboard civilian aircraft. The in-flight effective doses (route doses) estimated using actual flight path records in February 2026 were 51 μSv for the north route from Tokyo to Frankfurt and 30 μSv for the south route from Frankfurt to Tokyo. After conducting consistency checks of the calculated route doses using the in-flight measurement data obtained with an electronic neutron dosimeter, we have presented the effects of the changes in flight route (cut-off rigidity profile) on in-flight effective dose by comparing alternative route doses and great-circle route doses with an assumed constant cruising altitude (40,000 ft). The model-derived relative changes in the north, south, and round-trip route doses under the specified flight conditions between Tokyo and Frankfurt were +10%, −24%, and −6%, respectively. The effective dose per flight hour was notably reduced for the round trip along the alternative routes, implying that the annual dose of aircraft crew would decrease if their onboard working hours are carefully managed.

The better agreement was observed between the trends of the dosimeter-indicated values and the calculated H*(10) of HCPs (Figure 4B), rather than the total H*(10) values (Figure 4A). This finding is consistent with a possible sensitivity of the NRF31 to protons and helium ions, as implied from the neutron detection mechanisms shown in Figures 2C,D. The observed discrepancy between the calculation and measurement trends may have been partially caused by local shielding conditions, as the dosimeter was stored in a hand luggage placed beneath a passenger seat, which does not meet the standard reference conditions for either H*(10) or *Hp*(10) measurements. However, detailed quantitative discussions on the reason for the observed discrepancy are currently difficult owing to the lack of reliable data on dosimeter responses to some of the major cosmic radiation components at aviation altitudes, such as high-energy (>15 MeV) neutrons, protons, and helium ions, whereas it has been reported by the manufacturer that the NRF31 has a *Hp*(10)-based uncertainty of ±50% in neutron measurements at a similar dose level (32). To resolve this issue, we plan to conduct a comprehensive analysis of the dosimeter response using Monte Carlo simulations in future work, after obtaining the detailed technical information from the manufacturer (Fuji Electric Co., Ltd.), including its material composition, sensor geometry, converter structure, pulse-height processing, and neutron/HCP discrimination algorithms.

In the comparison with a previous study (21), our estimates of in-flight H*(10) values (57 μSv for the north route and 34 μSv for the south route) were within the range of the H*(10) values reported previously for similar routes between Tokyo and Paris: 56.2–74.1 μSv for the north route and 26.7–35.3 μSv for the south route. In addition to the inherent systematic errors that can be attributed to the different calculation models (34), the reason that our estimate for the north route is at the lower end of this range (56.2–74.1 μSv) can be partially explained by the difference in solar activity, as seen in the trend of heliocentric potentials (HPs) shown in Figure 6 (35). The previous study selected a period of solar minimum (March 2021) (21) when the HP value was 267 MV, whereas our study selected the solar maximum (February 2026) when the HP value reached 1,069 MV, a period during which more intense solar modulation deflected galactic cosmic-ray particles more strongly from entering the Earth’s atmosphere. The force field potential values used in our calculations using PARMA (25, 26) for both periods (March 2021 and February 2026) were 327 MV and 1,310 MV, respectively. The south route doses cannot be directly compared with the previously reported values because this route is affected by the recent onset of the conflict in the Middle East.

**Figure 6:**
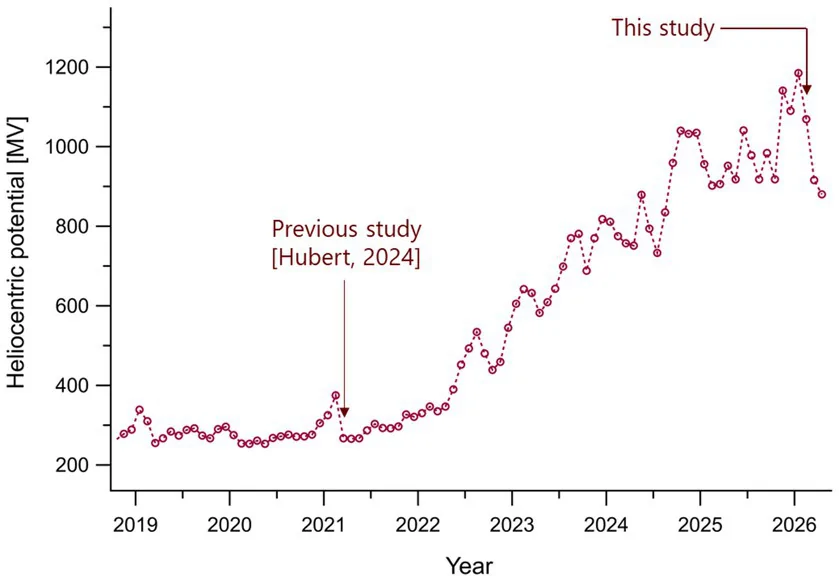
Temporal variations in heliocentric potential from 2019 to 2026 (35). The time period evaluated in a previous study (March 2021) (21) and that in this study (February 2026) are indicated on the plot. The United States Federal Aviation Administration (FAA) recommends using heliocentric potential values measured within two months of the target flight date for in-flight dose calculations using their CARI program (35).

The north and south route effective doses for flights with a constant cruising altitude (40,000 ft) between Tokyo and Frankfurt in February 2026 were calculated as 55 μSv and 34 μSv, respectively. Compared with the virtual flights along the great-circle route, the effective dose for the north route was 10% higher, and that for the south route was 24% lower. Consequently, the total effective dose (89 μSv) for the round trip along the alternative routes was just 6% lower than that (95 μSv) along the great-circle route. Although there is an inherent uncertainty in in-flight dose calculations (30), these model-derived relative changes (+10%, −24% and −6% for the north, south, and round-trip routes, respectively) represent ratios of effective doses calculated using the same code, the same solar-activity input, and the same altitude assumption. Consequently, the dominant systematic error components are common to both the numerator and denominator and largely cancel out. Our results suggest a limited effect of recent flight changes between Asia and Europe on cosmic radiation exposure of aircraft crew and passengers.

However, vulnerability to a solar particle event (SPE) is substantially higher on the alternative north route compared to the previous great-circle route. As shown in Figure 1C, aircraft taking the north route fly over the polar region with 0 GV cut-off rigidity for approximately 4 hours, whereas the minimum cut-off rigidity along the great-circle route does not fall below 0.6 GV (Figure 5A). The absence of cut-off rigidity, that is, the loss of geomagnetic shielding, implies that the current north route carries a potential risk of elevated radiation exposure from solar energetic particles (21, 30, 36, 37). This situation requires critical consideration from a radiological protection perspective. Comprehensive analyses of SPE doses under various conditions should be conducted in future studies, alongside validation based on continuous and extensive in-flight measurements.

This study has some limitations. Here, the route doses were presented for only one round trip from Tokyo to Frankfurt, which has the largest number of Japanese passengers among Japan–Europe flights. The effects of route changes could be larger for other flight routes that are affected more significantly by ongoing conflicts. Besides airspace restriction due to permission status, international flight routes can be changed due to various factors that affect the safety, cost, and schedule reliability, such as weather (38), air-traffic condition (39), pandemic outbreak (40), operational cost, and other unexpected events (41, 42). It is also desirable to monitor and evaluate the effects of flight route changes owing to these factors.

We used a commercial electronic dosimeter to check the consistency of calculated in-flight dose profiles because of its safety, portability, and ease of handling. However, its response to the complex mixed radiation fields at aviation altitudes, comprising high-energy protons and neutrons, remains highly uncertain and needs to be fully characterized to validate the calculated route doses. In addition, the dose contribution of helium ions, which has not yet been verified through comprehensive in-flight measurements, should be quantified. Furthermore, the possible effects of varied shielding conditions in an aircraft should also be examined using two or more dosimeters, as previous in-flight measurements using small dosimeters exhibited a dose variation depending on the location in the same aircraft (20). These potential uncertainties must be addressed and reduced in future studies.

## 5 Conclusion

In this study, we presented the in-flight effective doses on long-haul flights between Tokyo and Frankfurt, of which the flight routes have been significantly changed owing to the recent conflicts between Ukraine and the Russian Federation and in the Middle East. The effective dose for the outbound north route was higher than that for the previous great-circle route, and the inbound south-route dose was notably lower than the great-circle route dose. As a result, the total round-trip effective doses remained nearly unchanged. The results demonstrate that recent route changes have substantially altered the patterns of in-flight cosmic radiation exposure. Under normal conditions, overall changes in the total effective dose for these alternative routes may be small, and annual doses of aircraft crew may even decrease if their onboard working hours are carefully managed. However, the alternative north route carries a higher potential risk of elevated radiation exposure in the event of a solar particle event (SPE). Further comprehensive analysis of other flight conditions affected by ongoing conflicts and other factors is required to confirm the general applicability of the findings in this study.

## Data Availability

The original contributions presented in the study are included in the article/supplementary material, further inquiries can be directed to the corresponding author.
